# Supplementary material for: Add drop multiplexers for terahertz communications using two-wire waveguide-based plasmonic circuits
Source: Nat Commun. 2022 Jul 14;13:4090. doi: 10.1038/s41467-022-31590-z (PMC9283530; doi:10.1038/s41467-022-31590-z)
Supplement: Supplementary file 1 — Supplementary Information [file 41467_2022_31590_MOESM1_ESM.pdf]

Add drop multiplexers for terahertz communications using two-wire waveguide-based plasmonic circuits

YANG CAO[1], KATHIRVEL NALLAPPAN[1], GUOFU XU[1], AND MAKSIM SKOROBOGATIY[1,*]

[1]*Department of Engineering Physics, École Polytechnique de Montréal, Montreal, QC H3T 1J4, Canada*
**maksim.skorobogatiy@polymtl.ca*

Supplementary Note 1: Further discussion of the novelty and key contributions of this work

1. Components based on the two-wire plasmonic waveguides

High material absorption of most common materials in the THz band, as well as high waveguide dispersion, poses unique challenges for the development of THz waveguides capable of signal transmission with low insertion loss and distortion. Compared to the more common hollow metallic THz waveguides and dielectric THz fibers, the air-core two-wire plasmonic waveguides feature outstanding performance (low-loss, low-dispersion), ease of access to the modal fields, and ease of 2D and 3D on-chip integration, thus providing a promising platform for building THz circuits. The THz surface plasmon polariton (SPP) wave can be efficiently excited by the conventional linearly polarized THz source and guided in the air between the wires with negligible loss and dispersion. Originally, the two-wire waveguides were realized using the actual metallic wires. However, due to the engineering difficulty of precise aligning and keeping the constant separation between the two wires, the resultant devices were bulky and complex. As a result, mostly the simplest components capable of basic guiding THz light over a relatively short distance were realized [1]. Somewhat more complex devices based on the two-wire waveguides were demonstrated by inserting free-standing elements in the inter-wire air gap (ex. two-wire waveguide-based antennas [2] and waveguide Bragg gratings (WBGs) [3]), while still featuring limited structural stability and reliability in practical applications. In this work, we show that by resorting to additive manufacturing one can fully exploit the advanced optical characteristics of the two-wire waveguides. More precisely, we demonstrate a scalable strategy for the manufacturing of two-wire waveguide-based THz circuits based on standard optical elements such as WBGs, directional couplers, and Y-splitters. We also detail how such optical elements can be realized using two-wire waveguides augmented with some freeform elements. To our knowledge, this is the first time that the integrated optical circuits based on the two-wire waveguides are reported.

2. Fabrication route for the integrated terahertz circuits

In our work, we pursued a combination of 3D printing and metallization techniques as an alternative to CNC machining and microfabrication for the fabrication of THz plasmonic components. In addition to being cost-efficient and requiring relatively cheap infrastructure, this fabrication route allows innovative 3D waveguide and packaging designs, as well as integration of the freeform elements, thus capable of realizing plasmonic components that are far more advanced than classic two-wire or parallel-plate waveguides [4]. Compared with the silicon wafer-based integrated optical circuit whose template preparation requires costly top-of-the-line fab infrastructures, thus developed two-wire THz plasmonic circuit is easier to enter into production with competitive unit price. Furthermore, terahertz components demonstrated in this work feature inherently modular design with the waveguides, packaging, and alignment elements all integrated into the same component in a single fabrication step. This modular architecture allows trivial assembly and sectional reconfiguration of complex terahertz circuits without the need for painstaking alignment of the individual components. In contrast, to divert THz signal in system containing the existing integrated solutions, one has to replace the whole silicon chip and then set up its new optical path. Moreover, ease of access to the modal fields in the inter-wire air gap opens new ways of dynamic tuning of the optical component performance via various means, which is challenging for the existing silicon wafer-based integrated solutions due to their fixed planar structure and tightly confined modal fields.

Finally, our fabrication strategy is largely agnostic to the geometrical complexity of the optical elements, which opens a route for the integration of various freeform structures and the possibility of a truly 3D component integration. For example, strong Bragg gratings integrated with two-wire waveguides are demonstrated for the first time in this work and are made possible by augmenting a two-wire waveguide with a freeform periodic cascade of truncated cones. It is important to mention that the key limitation on the quality of optical components produced by the SLA printing technique is the printer resolution. As most budget printers feature a resolution of ~50 microns, terahertz circuits produced by such systems are limited for operation in the lower part of the THz spectrum. However, current advances in the SLA printing hardware and photosensitive resins already demonstrated commercial systems with resolution under 10 microns, with the only limitation being the high price of such systems. We, therefore, believe that the SLA printing technique will soon be able to create integrated photonic circuits for applications anywhere in the whole THz frequency range.

3. Two-wire add-drop multiplexers (ADMs)

To date, free-space ultra-high bit rate data transmission (> 100 Gbps) using a single-channel THz link has been reported [5-7]. Enabled by THz ADMs, the THz frequency division multiplexing is expected to multiply the data-throughput capacity of the THz communication networks. However, the development of the THz ADMs is still in the early stage with only a few examples known to date, such as those based on parallel metal plates or silicon waveguides and adapting component-design concepts that are commonly used in electronics or optics (ex. leaky-wave antenna, directional coupling) (see Table S1) [8-13]. Most reported optical-based THz ADMs are built on planar silicon substrate operating at THz frequencies ~300 GHz and having bandwidths of ~10 GHz. While functional, integration of the abovementioned ADMs into a complete THz system seems challenging as they require precise optical alignment, while some use bulky free-space components in their structure.

In our work, we report the four-port THz ADM circuits capable of channel dropping and adding the individual few-GHz-wide channels at ~150 GHz carrier frequency. In contrast to the prior works, we proposed a scalable and universal solution for the design and fabrication of the integrated signal processing THz circuits with additional advantages of the modular and reconfigurable design, as well as the cost-effective fabrication.

In summary, tandem ADMs that allow multiple THz signals at different carrier frequencies to be selectively dropped, added, or guided through are the key enabling element of the upcoming terahertz communications networks. We believe that our integrated circuits based on 3D printed two-wire waveguides could offer a pathway for the scalable manufacturing of such devices. In the future, unlike existing integrated solutions in a compact and fixed planar structure for universal terminal equipment, the two-wire

THz plasmonic ADM circuit can find applications in size-insensitive networks requiring customizable and reconfigurable configuration by taking advantage of its unique characteristics.

Table S1 Reported THz multiplexers for frequency-division multiplexing communications

Design	Dimensions	Operation principle	Functionality	Performance (Insertion loss & Bandwidth)	Advantages	Limitations	References
**Deformed PPWG**	3D<br>On the order of cm^3	Leaky-wave radiation through slot on a plate	Add/Drop	> 12 dB with tunable bandwidth for Add/Drop action	✓ Broadband operation frequency<br>✓ Tunable bandwidth	▫ Complicated optical alignment<br>▫ Compromise between bandwidth and crosstalk	*Nature Photonics,* 2015, 9(11) [8]<br>*Nature Communications,* 2017, 8(1) [9]
**PPWG & Electrically actuated components**	3D<br>On the order of cm^3<br>For 100 GHz	Coupling between waveguides with the assistance of resonance in cavity	Drop<br>&<br>Through	2.5 dB with a bandwidth of ~10 GHz for Drop action<br>&<br>2 to 6 dB within a broad band for Through action	✓ Relatively high efficiency<br>✓ Switchable output port for dynamic band allocation	▫ Complicated structure due to the use of active element<br>▫ Jagged spectral response for Through action	*Nature Communications,* 2018, 9(1) [12]
**Photonic crystal waveguides on silicon wafer**	2D<br>Less than 1 mm^2<br>For the key elements at 300 GHz	Directional coupling between adjacent waveguides<br>&<br>Photonic bandgap effect	Drop<br>&<br>Through	6.2 dB with a bandwidth of 6.5 GHz for Drop action<br>&<br>~6 dB with a bandwidth of 25 GHz for Through action	✓ Small size<br>✓ Possibility of tandem connection on a wafer	▫ High group velocity dispersion<br>▫ Narrowband with restricted spectral range for both Drop and Through actions	*Optics Express,* 2016, 24(7) [10]<br>*Advanced Optical Materials,* 2018, 6(16) [11]
**Unclad planar silicon waveguide**	2D<br>4 cm^2<br>For 300 GHz	Leaky-wave radiation<br>&<br>Constructive interference	Drop	~3 dB with a bandwidth of ~15 GHz for Drop action	✓ Supporting multiple channels<br>✓ Low insertion loss	▫ Complicated design<br>▫ Crosstalk between channels	*Optica*, 2021, 8(5) [13]
**Two-wire waveguide**	3D<br>0.33 cm^2×3.5 cm<br>For a key element (Grating-loaded side coupler, area inside its resin enclosure)<br><br>1.43 cm^2×12.5 cm<br>For full circuit having additional elements for optical characterization<br><br>For 100 GHz	Bragg reflection<br>&<br>Directional coupling	Drop<br>&<br>Add<br>&<br>Through	>4 dB for the key element (plus ~10 dB for beam steering in full circuits) within a bandwidth of <12 GHz for Drop & Add action<br><br>~6.5 dB for the key element (plus ~4 dB for beam steering in full circuits) within broadband for Through action	✓ Modular element for reconfigurable circuit<br>✓ Easy to align with other devices and possibility of tunable action<br>✓ Support of other modulation modalities (ex. Polarization Division Multiplexing) in a single device	▫ Large size of the full circuit<br>▫ Additional beam steering components are required for optical characterization<br>▫ High transmission loss of the currently employed beam steering solution	This work

Supplementary Note 2: Alternative fabrication routes

All-metal structures can be, in principle, an interesting alternative to the resin-based structures used in this work. From the fabrication point of view, however, partial metallization of 3D-printed plastic support is considerably easier than CNC machining of complex 3D circuitry from a metal slab. This is due to deeply sub-mm features of the waveguide support and grating structure, as well as the 3D complexity of the design. Alternatively, one might attempt fabrication of the all-metallic structures using additive manufacturing approaches such as selective sintering of metallic powders. To date, however, the resolution of the metal powder-based printers is in the 100-250 um range, which is much less performant than the 10-50 um resolution of the resin-based SLA printers.

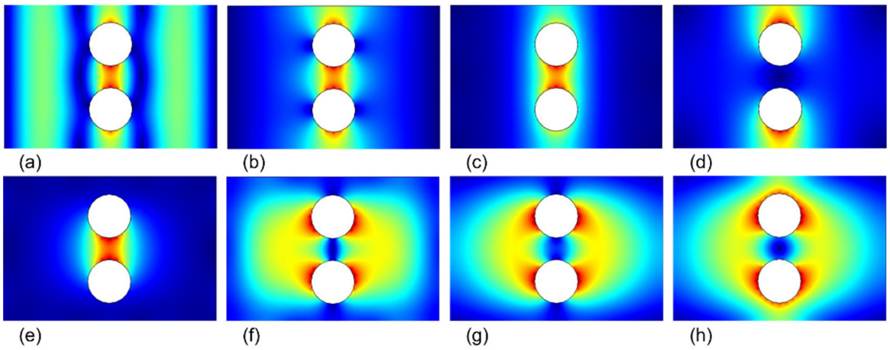

Fig.S1 Numerical simulation on micro-encapsulated two-wire waveguide in different designs. Electric field distributions of several lowest-loss plasmonic modes at 140 GHz supported by a hollow enclosure with metallic walls (a rectangular waveguide) containing two metallic wires (a) mode 1 (loss 1.6 m^{-1}), (b) mode 2 (loss 2.2 m^{-1}), (c) mode 3 (loss 2.4 m^{-1}), (d) mode 4 (loss 2.6 m^{-1}). Electric field distributions of several lowest-loss plasmonic modes at 140 GHz supported by an identical hollow enclosure with infinite plastic walls containing two metallic wires (e) mode 1 (loss 5 m^{-1}), (f) mode 2 (loss 45 m^{-1}), (g) mode 3 (loss 52 m^{-1}), (h) mode 4 (loss 68 m^{-1}). The loss is by field.

Additionally, all-metallic encapsulation of the two-wire waveguides can pose significant challenges due to the presence of a large number of higher-order, relatively low-loss modes that can cause multi-mode interference effects and bring about additional noise and artifacts to the operation of an ADM circuit. This is because a hollow metallic encapsulation cage is essentially a heavily multimode rectangular waveguide that supports a large number of relatively low-loss modes even when two metallic wires are placed inside. In Figs. S1(a-d) we present several of such plasmonic modes and their losses computed using the Impedance Boundary Conditions (see Methodology) for all the metallic walls. From this calculation, we see that for waveguides shorter than 1 m, all these modes will persist inside the waveguide and cause multimode interference. In comparison, by substituting metallic walls with the infinite resin ones (see Figs. S1(e-h)), we observe that only a single mode with the highest field concentration in the gap between the two wires has a relatively low loss, while all the other modes have much higher losses. Thus, using a resin cage in place of a metallic one reduces the number of low-loss higher-order modes, thus suppressing the multi-mode interference effects, resulting in essentially single-mode operation of an encapsulated two-wire waveguide.

For these reasons, we believe that partial metallization of the 3D-printed resin-based structures is currently the best option for the fabrication of complex plasmonic THz circuits.

Supplementary Note 3: Tunability of the 3D printed two-wire plasmonic waveguide components

Generally, tunable ADMs can be of great benefit for applications in dynamic bandwidth allocation, which, in practical terms means the ability to change the ADM frequency response. Considering that the two-wire waveguide-based ADM circuits presented in this work are built on a 3D printed resin substrate, the thermal and mechanical tunability is available by changing the physical properties and geometry of the resin substrate. Thermal tuning is possible by changing the temperature of the frequency-dependent element, i.e., the WBG of an ADM. Furthermore, mechanical tuning of an ADM presented in this work is possible using the grating-loaded side coupler built on a compressible dielectric support. Under axial strain, the deformation would result in the change of a grating period, and as a consequence, a shift in the Add/Drop frequency. Practically, this can be realized by using a variety of commercial photosensitive resins featuring large thermal expansion coefficient (ex. shape-memory polymer) or low Young's modulus value (ex. Monocure FLEX100 Resin) when 3D printing the ADM structure. The circuit switching time will depend crucially on the tuning mechanism. For thermal tuning, the switching time is typically in seconds, while for mechanical tuning it can be in milliseconds. If faster switching times are needed, one normally resorts to optical or electric tuning.

That said, the slow response of the thermo-mechanical actuation impedes its application for true dynamic bandwidth allocation, while it is certainly suitable for applications in quasi-static switching. In this respect, the classic two-wire metallic waveguides cannot compete with a silicon platform that profits from the possibility of ultra-fast charge injection tuning. That said, several routes can be pursued to make charge injection tuning possible in the case of two-wire waveguides. One is deposition on top of metallic layers of a semiconductor material (ex. InSb, etc.) that features plasma frequency in the near THz frequency range [14]. In such materials, the plasmonic frequency can be readily changed via carrier injection, thus altering the optical response of a waveguide. Another is the insertion of an optically or electrically tunable film directly into the air gap of a two-wire waveguide. By changing the conductivity of such a film, one can realize phase and amplitude modulation of the transmitted THz mode. Particularly, in the case of graphene sheets, their complex conductivity can be adjusted on a timescale of picoseconds by changing its Fermi energy via optical pumping or electrical gating [15-17], while their ability to modulate THz wave propagating in a two-wire waveguide was recently demonstrated in [18,19]. We, therefore, believe that the integration of active materials into the two-waveguide structure can make such waveguides tunable both optically and electrically.

Therefore, we believe that in contrast with a silicon platform that features a fixed planar structure and tightly confined modal fields, the air core two-wire waveguides feature a highly flexible 3D structure and easily accessible modal fields, thus allowing a variety of tuning modalities ranging from the relatively slow thermal and mechanical actuation to ultra-fast optical and electronic tuning.

Supplementary Note 4: Design of the two-wire waveguide Bragg gratings

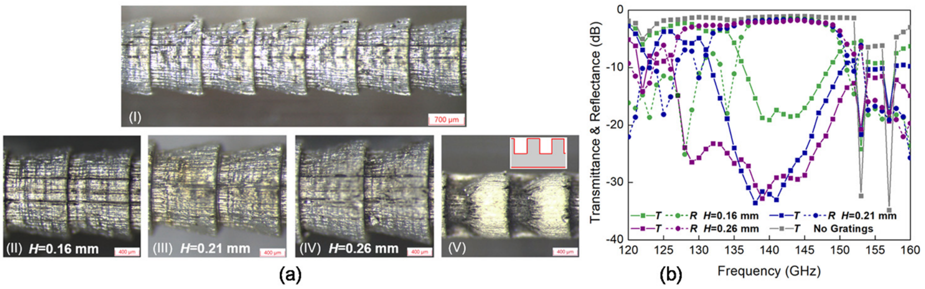

Fig. S2 Design of two-wire WBGs. (a) (I) Photographs from the top of a fabricated WBG with a period of Λ=1.03 mm and a ridge height of H=0.21 mm (only the WBG half is shown). (II-IV) Enlarged view of the metalized gratings (truncated cones) of different ridge heights H. (V) Photograph of a metalized grating featuring rectangular grooves and its schematic. (b) The numerical transmittance and reflectance (by power) of the 2.5cm-long WBGs containing 20 periods with Λ=1.03 mm as a function of the ridge height H.

The two-wire WBG features periodic variation in cross-sections of one wire, while the other wire is uniform and has a fixed diameter of D=1.321 mm. The periodic structure is a sequence of end-to-end connected truncated cones added on top of a uniform circular wire of diameter D (see Fig. S2(a) (I-IV)). The choice of truncated cones in the grating structure was experimentally found to be the most reliable and stable for printing compared to other alternatives such as rectangular or sinusoidal ridges, or rectangular grooves on the wire surface. The deformations in these 3D printed structures are mainly attributed to the "cure-through" effect during 3D printing. Particularly, the UV radiation not only cures the resin within the top printed layer but also leaks through the cured layer and solidifies some resin on the other side. This uncontrollable effect occurs repeatedly, thus resulting in the cumulative deformation of prints, which is most pronounced when the geometry changes rapidly from one layer to another. For example, the ideal steep slope at the edge of a rectangular ~600um-deep groove engraved on a wire was deformed into a conical structure when printed (see Fig. S2(a) V). This problem highlights the importance of carefully choosing the photosensitive resin chemistry to match the UV absorption depth with the desired layer thickness. Finally, we mention that gratings used in this work can only be printed along a single direction from the cone smaller base towards its larger base to avoid overhanging structures.

In Fig. S2(b) we show computed transmittance and reflectance of THz light propagating through 2.5cm-long WBG sections as a function of the ridge height H, which effectively controls grating strength (stopband bandwidth). The gratings contain 20 periods of truncated cones with a grating period of Λ=1.03 mm. As a reference, transmittance for a straight two-wire waveguide of the same length is also shown. Due to the anticrossing of the waveguide fundamental modes with those of a resin cage, sharp dips in the reference waveguide transmission spectrum are present at 122 GHz and 155 GHz, while in-between these values waveguide transmission is relatively featureless. A similar anticrossing phenomenon is also observed numerically in the transmission spectra of WBGs. However, such spectral features are very sensitive to surface roughness and other structural imperfections and are not easily observed experimentally as reported earlier in Ref [4]. We also note that increasing the ridge height H from 0.16 mm to 0.26 mm results in a strong increase in the size of the grating stopband from ~12 GHz to ~25 GHz calculated as the full width at half maximum (FWHM) of the reflectance curve. Additionally, an increase in the ridge height results in a smaller average gap size between the two wires, and as a consequence, the higher effective refractive index of a WBG mode, thus reducing the center frequency of the grating stopband. Considering the finite transverse resolution of the stereolithography (SLA) 3D printer (50 µm in our case), the truncated cone-shaped grating section featuring larger ridge heights (Fig. S2(a) IV) can be fabricated easier and more reliable than those with smaller heights (Fig. S2(a) II). However, at the same time, larger ridge heights require tighter tolerances for the various sizes of the supporting cage due to the smaller separation between the grating wire and the uniform wire. Using larger ridge heights also tends to result in lossier gratings due to the stronger scattering effect of imperfections in the grating structure on the guided modal fields. Therefore, an optimal ridge height of the gratings must be chosen to ensure large operational bandwidth, reproducible optical performance, and manageable loss. In this work, by comparing optical properties of several WBGs we find the optimal ridge height to be H=0.21 mm (see Fig. S2(a) I and III). With the period of a WBG set to Λ=1.03 mm, the resultant stopband center frequency is 140 GHz with the corresponding bandwidth of ~18 GHz.

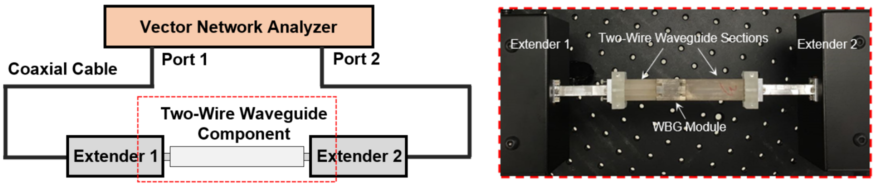

Fig. S3. Schematic of the network analyzer to characterize THz two-wire waveguide components. The red dotted box shows the connection between two-wire waveguide components and extender modules via rectangular waveguides.

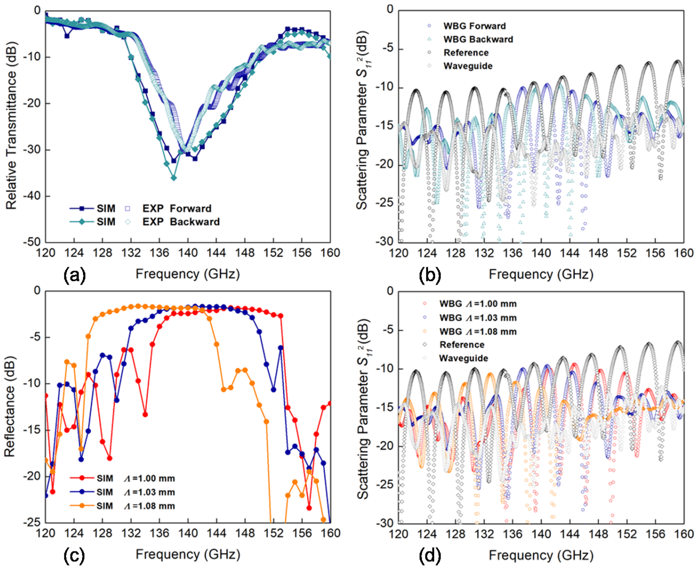

Fig. S4 Two-wire waveguide Bragg gratings. (a) Relative transmittance and (b) Measured scattering parameter (S_{11}^{2}) of a 2.5cm-long WBG section comprising 20 periods (Λ=1.03 mm, H=0.21 mm) for THz light with two possible traveling directions. (c) Reflectance and (d) Measured scattering parameter (S_{11}^{2}) of 2.5cm-long WBGs containing 20 periods (H=0.21 mm) as a function of the period length Λ. The THz light travels in the forward direction along WBG (see Fig. 2(a)).

The two-wire WBGs were studied using a network analyzer with extender modules (frequency multipliers) (see Fig. S3). Although the proposed WBG exhibits nonsymmetric structure (see Fig. 2(a)), it, nevertheless, features similar relative transmittances for the THz light traveling along either of the two directions along WBG (see Fig. S4(a)). Particularly, Fig. S4(b) shows measured reflectance (S_{11}^{2}) for a WBG connected to the extender module via a two-wire waveguide section. The standing waves caused by the parasitic reflections between the WBG and the detector greatly complicate the interpretation of the measured results due to periodic ripples in the measured spectra. In the absence of THz isolators, selective attenuation of the parasitic back-reflected waves is challenging (if not impossible). While the reference reflectance spectrum can be recorded by placing a planar metallic mirror at the output plane of the two-wire waveguide section (with WBG removed), it is still not trivial to remove oscillations from the WBG reflected spectrum. This is due to the difference in the effective light travel distance in the WBG/waveguide and mirror/waveguide (reference) measurements. Nevertheless, when comparing the normalized reflectance values of the WBG to those of numerical simulations, a good comparison is found (see Fig. 2(d)). In addition, by varying the grating periodicity of WBGs, spectral positions of the reflection peaks, as well as bandwidths of the reflection spectra, agree well with the theoretical predictions (see Figs. S4(c)and S4(d)).

Supplementary Note 5: Y-splitters used in the directional coupler and the ADM circuits

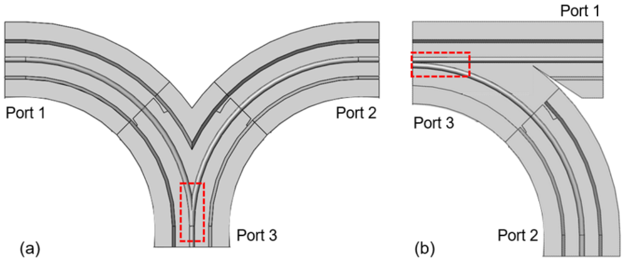

Fig. S5 Schematics of (a) Y-coupler and (b) Y-splitter (top part removed). The red dotted regions show Y junctions in each component.

In our work, we use both a Y-splitter and a Y-coupler. While the two devices sound the same their function and operation are different. Thus, a Y-coupler (see Fig. S5 (a)) features two physically coalescing bent waveguides. Thus, when launching light into port 3, it will be equally split into port 1 and port 2. In this work, the Y-coupler is used to measure the WBG reflectance (see Supplementary Note 9). There, the light is launched into port 1 and collected at port 2, while the THz signal is back-reflected at port 3 either from the WBG or the reference mirror [4]. Note that Y-couplers bring an additional 3 dB loss after reflection at port 3 and equal splitting into ports 1 and 2, thus lowering the SNR of the measurement.

In contrast, a Y-splitter used in the ADM circuits for beam steering (see Figs. 1 and 3(b)) is different from the Y-coupler both in geometry and in function. As seen from Fig. S5 (b), the two waveguides in the splitter (one straight, another bent) physically coalesce. The Y-splitter is designed so that its cross-section at port 3 is identical to that of a directional coupler of the ADM circuit. As a result, at port 3 there is not one, but two physically distinct waveguides (unlike in the case of a single waveguide at port 3 of a Y-coupler), and there is no additional 3 dB loss incurred at port 3 of a Y-splitter. In fact, the leading loss mechanism of a Y-splitter is simply related to the propagation losses of the straight and curved waveguides of the two branches. Finally, we note that due to mismatch in the modal field distributions of the straight and curved waveguides of otherwise identical cross-sections, one also expects an additional small insertion loss at the coupling plane between a side coupler and a Y-splitter (of identical cross-sections*)*.

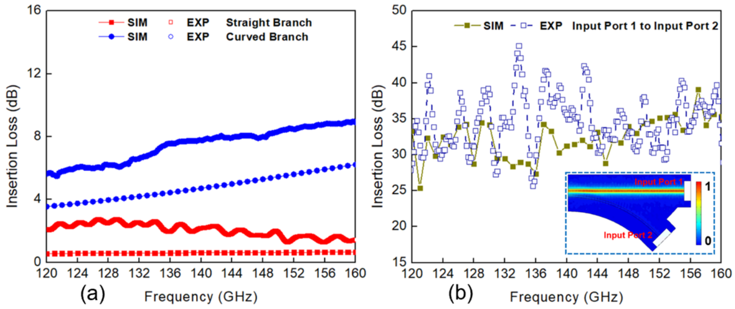

Fig. S6 Two-wire Y-splitters. (a) The insertion losses of the straight and curved arms of the Y-splitters in the directional coupler and ADM circuits for numerical and experimental studies. (b) The insertion loss of the THz light launched into Input port 1 and registered at Input port 2 of Y-splitters. Insert: the computed electric field distributions in the Y-splitter when using the fundamental mode of Input port 1 at 140 GHz as the excitation condition in numerical simulations.

In this work, the two-wire waveguide-based wideband Y-splitter (see Figs.1, 3(b), 3(d), 4(a), and S5(b)) was used to guide THz signals to/from the side coupler section from/to the desired ports of the directional coupler and ADM circuits. It features a curved two-wire waveguide (circular arc featuring a radius of 4 cm) gradually approaching a 4.5cm-long straight two-wire waveguide while joining in a wedge configuration at the end. In this work, the side coupler sections were connected with Y-splitters at their two ends to form the complete directional coupler and ADM circuits. In the numerical simulation, the insertion losses of straight and curved two-wire waveguides with the same length as the two branches of a numerical Y-splitter (see Fig. 4(a)) were computed using 2D and 2D axisymmetric mode solver tools of COMSOL Multiphysics, respectively (see Fig. S6(a)) [4]. In experiments, to remove coupling losses between extenders and the two-wire waveguides, which were connected using standard rectangular metallic waveguides, the transmission spectrum ($S_{ref,21}{}^2$) of a straight two-wire waveguide assembled by two waveguide sections was measured at first. After placing the straight or curved waveguide featuring the same length as one branch of an experimental Y-splitter (see Fig. 3(b)) between these two waveguide sections, the insertion loss of the inserted waveguide was then measured by comparing the recorded transmission spectrum ($S_{21}{}^2$) and the reference $S_{ref,21}{}^2$.

Additionally, Fig. S6(b) shows that in Y-splitter, the THz light that is launched into one port mostly propagates in the forward direction (larger than 30 dB by power) with only a minimal amount of crosstalk to the other port. Therefore, when using Y-splitters in directional coupler and ADM circuits, their contribution to the crosstalk is negligible.

Supplementary Note 6: Numerical simulations of the directional coupler circuits

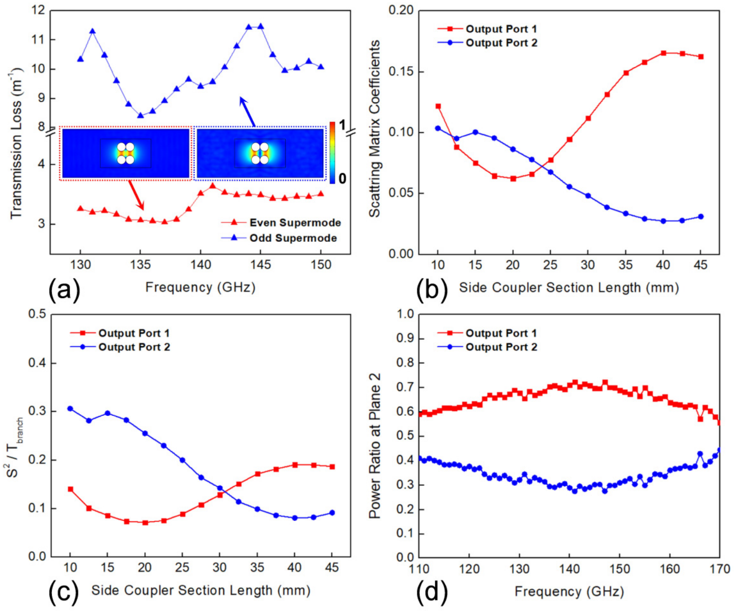

Fig. S7 Numerical simulation results of directional coupler circuit. (a) The transmission loss (by field) of the even and odd supermodes supported by the side coupler section that contains two two-wire waveguides. Insert: the modal electric field distribution of the two supermodes at 140 GHz operational frequency. (b) The numerically computed power transmittance at Output port 1 and Output port 2 assuming excitation at the Input port 1 of the directional coupler circuit (see Fig. 3(d)) as a function of the length of the straight side coupler section. (c) The numerically computed power transmittance of the two waveguides (Waveguides 1 and 2) at the end (Plane 2) of the side coupler section assuming excitation at Input port 1 of the directional coupler circuit (see Fig. 3(d)) as a function of the length of the straight side coupler section (obtained by using Eq. (1)). (d) The ratio of the power carried by the two two-wire waveguides (Waveguides 1 and 2) at the end (Plane 2) of a 40.6mm-long side coupler section when it is integrated into the directional coupler circuit shown in Fig. 3(d), whose value is computed using Eqs. (1) and (2).

Supplementary Note 7: Numerical simulation of the two-wire ADM circuits

In what follows we detail the design and optimization of the ADM circuit shown in Figs. 1 and 4(a). The orange arrows represent THz signals that are within the stopband of a grating, while green arrows correspond to those in the grating passband. The path of the THz light that is launched into In port (Waveguide 1) is marked by solid arrows, while that launched into Add port (Waveguide 2) is marked by hollow arrows. While propagating in the grating-loaded side coupler, the THz signal within the grating stopband that is launched into In port on Waveguide 1 (Add port on Waveguide 2) is back-reflected by the gratings, and then transferred to Waveguide 2 (Waveguide 1) by the coupler. The THz signal within the grating passband when launched into In port propagates through the grating-loaded side coupler and into Through port.

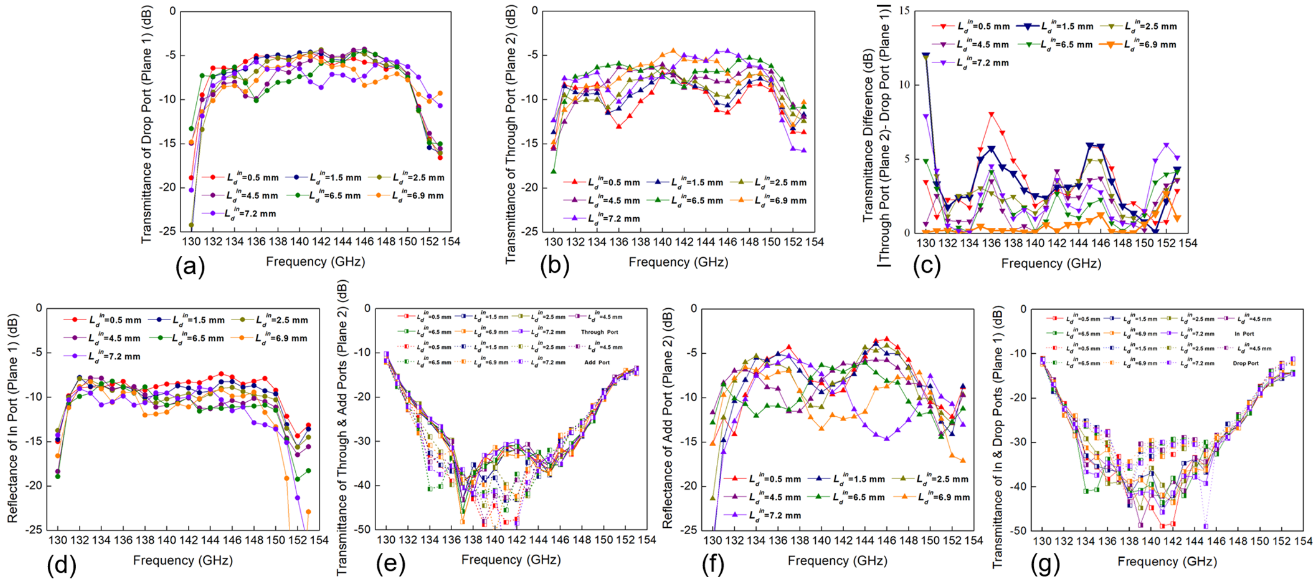

Fig. S8 Numerical simulation results of 35mm-long grating-loaded side coupler sections containing 20 periods with Λ=1.03 mm, H=0.21 mm, and different L_d^{in} computed using Eq. (1). Numerical transmittances (by power) at (a) Plane 1 for Drop port under In port launching, and (b) Plane 2 for Through port under Add port launching conditions. (c) Minimizing the difference between power transmittance of the Drop port at Plane 1 (Drop function) and that of the Through port at Plane 2 (Add function) to equalize the Add and Drop functions. Numerical reflectance (by power) at (d) Plane 1 for In port, and (f) Plane 2 for Add port of ADM circuits. Parasitic transmittance (by power) at (e) Plane 2 for Through and Add ports under In port launching (Plane 1), and (g) Plane 1 for In and Drop ports under Add port launching (Plane 2) conditions.

Firstly, we numerically study transmittance and reflectance (by power) characteristics of the 35mm-long grating-loaded side couplers containing 20.6mm-long gratings using Eq. (1) and scattering matrix coefficients (S^2) at the In, Drop, Add, and Through ports for various launching conditions. It is found that transmittance of Drop (Through) port under In (Add) port launching condition varies with the L_d^{in} parameter, thus confirming the designability of channel Drop (Add) response of ADM circuit. With proper designs, the channel adding and dropping of the proposed two-wire ADMs can be contiguously efficient within the whole grating stopband for broadband THz signal, while can also be selectively efficient at certain spectral position within the grating stopband for narrowband channels. In this work, particular attention was paid to the design of two optimal grating-loaded side couplers that enable the ADM circuits with optimized Drop and balanced Add/Drop actions, respectively.

Numerical simulations show that the maximum value of the transmittance of Drop port at Plane 1 for the operating frequency within the grating stopband ~ 140 GHz ± 6 GHz, which gives rise to the optimized Drop action of ADM circuit, is obtained when L_d^{in}~0.5-2.5 mm (see Fig. S8(a)). This shows the realistic tolerances when choosing the value of L_d^{in} for the optimized Drop action considering that the SLA printer resolution is ~50 um.

For the design of the other optimal ADM with balanced Add/Drop action, one needs to minimize the difference between transmission through the Through port under Add port launching condition and transmission through the Drop port under In port launching condition. Particularly, we vary the L_d^{in} parameter to minimize the absolute difference between transmittances of the Through and Drop ports (see Figs. S8(a-c)). Clearly, choosing identical left and right grating offsets $L_d^{in} = L_d^{out}$ should result in the balanced performance of channel dropping and adding in case of the ADM with symmetric gratings [20,21]. In our case, we observe that the balanced ADM performance is achieved when L_d^{out}- L_d^{in} = 0.6 mm (L_d^{in}=6.9 mm). This somewhat off-center positioning of gratings is attributed to the small asymmetry in the grating performance depending on the direction of propagation of light.

Additionally, we also present the analysis of parasitic transmissions into Through and Add ports (Plane 2) when light is launched into In port (Plane 1) within the grating stopband. In this case, most of the energy (~15-40%) is transferred into Drop port (Plane 1) (Fig. S8(a)), some is reflected into In port (Plane 1) (~5-15%) (Fig. S8(d)), while parasitic transmissions to Through and Add ports (Plane 2) constitute <1% (Fig. S8(e)). Similar conclusions are reached for the four ports when light is launched into Add port (Plane 2) within the grating stopband (see Figs. S8(b), S8(f), S8(g)).

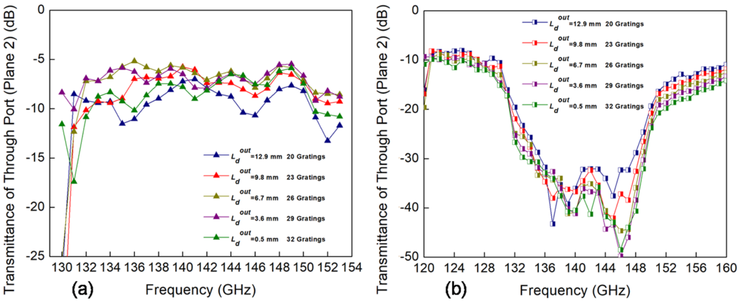

Fig. S9 Numerical simulation results of 35mm-long grating-loaded side coupler sections containing gratings featuring different numbers of periods with Λ =1.03 mm, H=0.21 mm, and L_d^{in} =1.5 mm computed using Eq. (1). Numerical transmittances (by power) at Plane 2 for Through port under (a) Add port launching (Plane 2), and (b) In port launching (Plane 1) conditions.

For the above-mentioned grating-loaded side couplers, the value of L_d^{out} might be suboptimal as it is not truly a free parameter. In fact:

$$L_d^{out} = L_{coupler} - L_d^{in} - N_g \Lambda \quad \text{(S1)}$$

Since L_d^{in} has been determined as 1.5 mm to enable the optimized Drop action, L_d^{out} can be adjusted by varying the number of grating periods to optimize Add action simultaneously. One usually can set $L_d^{out} \approx L_d^{in}$, which requires the use of longer gratings (ex. N_g=30) (see Fig. S9). However, we found experimentally that longer Bragg gratings N_g ~22-31 result in high transmission losses due to scattering loss on the imperfectly printed grating structure. At the same time, one requires at least N_g =15-20 in experiments to make sure that the grating length is longer than the light penetration depth into the grating L_g. Thus, for the ADM design with imperfect gratings, there is a tradeoff between the equivalently optimized performance of channel dropping and adding and the ADM loss. In experiments, we prioritized low ADM losses while somewhat sacrificing the optimized operation of both of Add and Drop ports by choosing N_g =20, which is mostly manifested by the elevated back reflection in the case of Add port (Plane 2) (see Fig. S8(f)) which is more pronounced than back reflection into In port (Plane 1) for the Drop action (see Fig. S8(d)).

Supplementary Note 8: Insertion losses of the ADM circuits

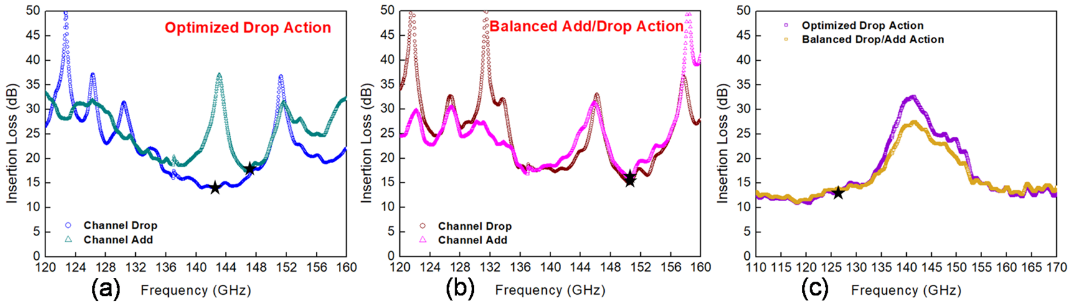

Fig. S10 The insertion losses of complete ADM circuits. The measured insertion losses (by power) under the In (Add) port launching conditions as registered at the Drop (Through) port (see Fig. 1) of the ADM circuit exhibiting (a) optimized Drop action, and (b) balanced Add/Drop action. (c) The measured insertion losses (by power) under the In port launching condition as registered at the Through port.

The insertion losses of the ADMs presented in this paper were computed by first measuring the transmission spectra (S_{21}^2) of different ports under the appropriate launch conditions. Such spectra were then normalized by the reference to remove coupling losses between extenders and the two-wire waveguides of the ADM circuits, which were connected using standard rectangular metallic waveguides. As a reference, we chose a transmission spectrum ($S_{r,21}^2$) of a straight two-wire waveguide section of length l as measured by the same spectroscopic system using the same rectangular waveguides for coupling. Finally, to remove the effect of a reference waveguide transmission loss $\alpha(\omega)$ (that was measured independently in [4] using a cutback method) we multiplied the reference spectrum by the factor of $\exp(\alpha(\omega)l)$. Thus, the ADM insertion loss with the coupling loss to the extenders removed was computed as:

$$\text{Insertion loss} = 10\log_{10}\left(\frac{S_{21}{}^2}{S_{r,21}{}^2\exp(\alpha(\omega)l)}\right) \tag{S2}$$

The insertion losses of THz light registered at different ports under In and Add port launching conditions are shown in Fig. S10. For the ADM with optimized Drop action (L_d^{in} =1.5 mm), the minimum insertion loss by power (within the Bragg grating stopband) as registered at Drop port under In port launching was measured as ~14 dB at ~143 GHz. While for the Through port under Add port launching, the minimal insertion loss was ~18 dB at ~148 GHz for channel adding (see Fig. S10(a)). Similarly, for the ADM circuit with balanced Add/Drop performance (L_d^{in} =6.9 mm), the minimal insertion losses were measured at two spectral locations ~138 GHz and ~151 GHz with the corresponding values of ~18 dB and ~15 dB for Drop port under In port launching and ~18 dB and ~16 dB for Through port under Add port launching. Besides, an insertion loss peak appears around the center of the grating stopband (see Fig. S10(b)). Finally, insertion losses for the two ADM designs as registered at Through port under In port launching are shown in Fig. S10(c). The pronounced loss peaks corresponding to the Drop action are clearly observable in the vicinity of ~140 GHz.

It is worth noting that such differences in the overall efficiency and bandwidth of channel Add/Drop and Through functions of the full THz ADM circuit can not diminish its value as a THz multiplexer for multi-channel communications. Indeed, the proposed full ADM configuration was designed for the convenience of characterizing its crucial element (i.e., grating-loaded side coupler section) by THz spectroscopy systems, not aiming at building the trade-off between channel Add/Drop and Through actions. Therefore, it is not surprising to observe such imbalances in our case, which is also common for mid-IR ADMs in telecommunication networks. For the proposed two-wire ADM, one can simply adjust the structure of its replaceable beam steering element (Y-splitters) and superimpose other grating structures (ex. uniform gratings with high index contrast (see Fig. S2(b)) on its indispensable directional coupler section to reduce the difference in the overall efficiency and bandwidth, respectively.

Supplementary Note 9: Characterization of the two-wire waveguide-based Bragg gratings and the ADM circuits using a free-space continuous-wave (CW) THz spectroscopy system

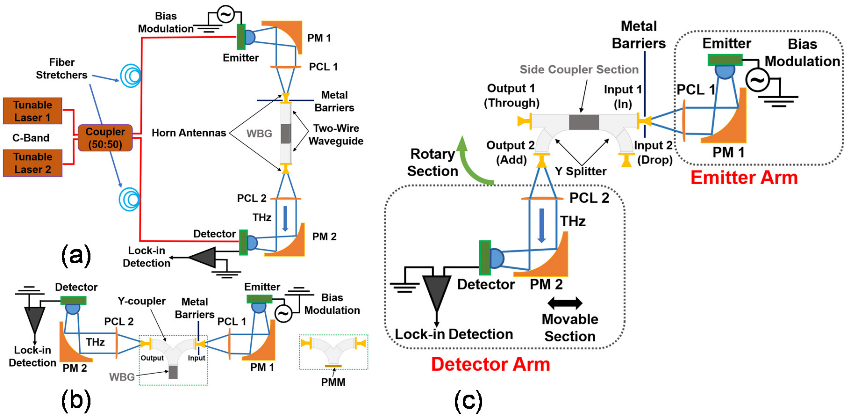

Fig. S11 CW-THz spectroscopy system. (a) Experimental setup to characterize the relative transmittance of two-wire WBGs. PM: parabolic mirror, PCL: plano-convex lens. (b) Experimental setup to characterize the reflectance of two-wire WBGs. Insert: the THz optical components to replace the ones that have been placed inside the CW-THz spectroscopy system (in green dotted box) for the reference spectrum. PMM: planar metallic mirror. (c) Experimental setup to characterize the ADM circuits.

The proposed THz two-wire waveguide-based components and circuits were also characterized using the free-space CW-THz spectroscopy system (Toptica Photonics). The schematic of the experimental setup is shown in Fig. S11 and briefly explained as follows. Two distributed feedback (DFB) lasers with the power of ~30 mW each, operating in the infrared C-band with slightly different emission wavelengths are used as the source of THz generation. A 50:50 fiber coupler is used to combine the two laser beams and split them into both emission and detection arms respectively. By applying AC bias voltage to the emitter photomixer, the THz radiation corresponding to the beat frequency between the two DFB lasers is generated. The output THz frequency can be varied by simply tuning the emission wavelengths of the lasers. A similar photomixer (without bias voltage) and a lock-in amplifier are used in the THz detection arm. The focused linearly-polarized CW-THz beam transmitting through the waveguide components or circuits is re-collimated and the amplitude of the THz signal is recorded using lock-in detection. The phase of the THz signal is simultaneously recorded by using the fiber stretchers of equal lengths that are connected to both emitter and detector arms. Using the experimental procedures that are similar to the ones with network analyzer which has been illustrated in Methodology, the relative transmittance of WBGs and transmittances of grating-loaded side couplers were characterized by CW-THz spectroscopy system and shown in Figs. S12 and S13. Compared with the ones that are shown in Figs. 2 and 5, it is found that these two sets of experimental results are consistent in general, which verifies the intended functionality of the proposed two-wire waveguide components and circuits. However, the standing waves, which are formed in the cavity of the CW-THz spectroscopy setup, as well as inside of a photomixer silicon lens, impose periodic ripples on the measured transmission spectra. Although such spectral oscillations can be somewhat mitigated via normalization with respect to a properly chosen reference, they are, nevertheless, notoriously difficult to eliminate (see Figs. S12 and S13) [22,23].

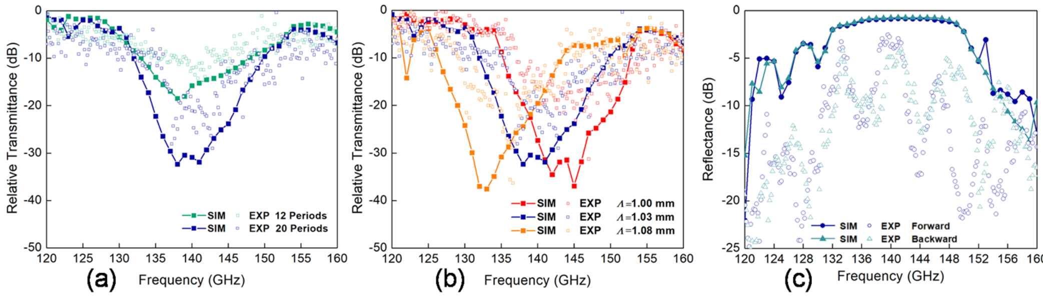

Fig. S12 Two-wire WBGs. The numerical and experimental relative transmittances (by power) of 2.5cm-long WBG sections (a) comprising different number of periods (Λ=1.03 mm, H=0.21 mm), and (b) containing 20 periods (H=0.21 mm) as a function of the period length Λ. The THz light travels in the forward direction (see Fig. 2(a)). (c) The numerical and experimental reflectance (by power) of 2.5cm-long WBG sections comprising 20 periods (Λ=1.03 mm, H=0.21 mm) with two possible traveling directions of the THz light.

The reflectance of WBG (see Fig. S12(c)) was characterized by the CW-THz spectroscopy system as follows. Firstly, the two-wire Y-coupler and the WBG were assembled using the interconnects at the end facets of both components. Then, two WR6.5 conical horn antennas were connected to the unused ports, namely input and output ports of the Y-coupler. The THz light was launched into the input port of the Y-coupler, guided through a curved arm of the coupler, and reflected from the WBG under study. The reflected light was then divided equally by the Y junction and directed towards both the input and output ports. The transmission spectrum of THz light at the output port was recorded. Next, the WBG was replaced by a planar metallic mirror to completely reflect the THz signal at the Y-junction (see Figs. S5(a) and S11(b)). The transmission spectrum recorded at the output port was used as the reference. Finally, the reflectance of WBG was obtained by comparing these two measured transmission

spectra. However, due to the standing waves formed in the cavity of the CW-THz spectroscopy setup and photomixer silicon lenses, the high insertion loss of the desired THz signal propagating through the assembled circuit, as well as the noise induced by the coupling between the two curved branches of Y-coupler, this measurement features low SNR, thus resulting in the experimental results differing from the numerical ones.

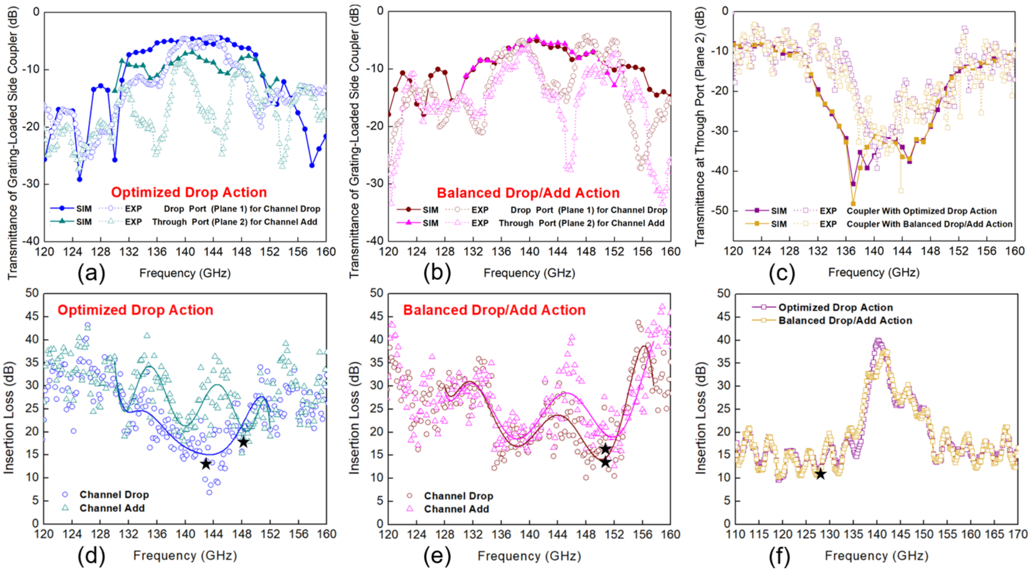

Fig. S13 Optimized grating-loaded side couplers. Power transmittances of the grating-loaded side couplers for (a) an ADM with the optimized Drop action (L_d^{in}=1.5 mm) and (b) an ADM with the balanced Add/Drop action (L_d^{in}=6.9 mm) under the In port launching (Plane 1) and the Add port launching (Plane 2) conditions. (c) Power transmittances of the two grating-loaded side couplers at the Through port (Plane 2) under the In port launching (Plane 1) condition. When integrating the complete ADM circuit into two-wire THz waveguide-based communication networks, the measured insertion losses (by power) under In (Add) port launching conditions as registered at Drop (Through) port of the complete ADM circuit (see Fig. 1) exhibiting (d) optimized Drop action, and (e) balanced Add/Drop action. Solid lines are the 8th-degree polynomial fits. (f) The measured insertion losses (by power) under In port launching condition as registered at Through port.

Supplementary Note 10: Characterization of the directional coupler circuits using a THz communication system

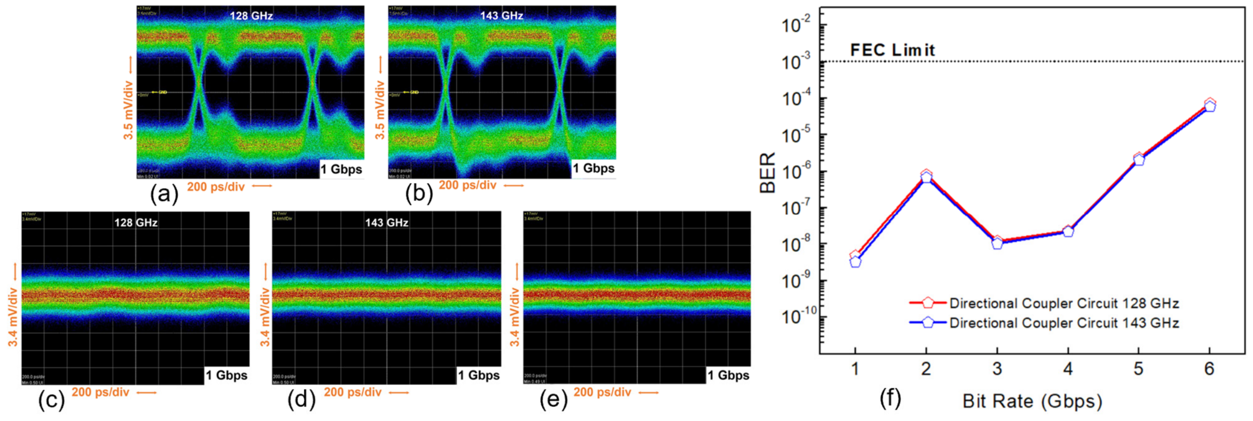

Fig. S14 Experimental study of the directional coupler circuit shown in Fig. 3(b) for THz communications. Eye pattern of the THz signal that is launched into the Input port 1 and registered at the Output port 1 with the carrier frequency of (a) 128 GHz and (b) 143 GHz. Eye pattern of the THz signal which is launched into the Input port 1 and registered at the Input port 2 with the carrier frequency of (c)128 GHz and (d) 143 GHz. (e) Eye diagram for the noise level of the THz communication system (no signal). (f) Measured BER versus bit rate for the THz signal propagating through the directional coupler circuit at 128 GHz and 143 GHz carrier frequencies.

To verify that the Add/Drop functionality of the ADM circuits presented in this work is due to Bragg gratings, we conducted a comparative study of a stand-alone directional coupler circuit (without a grating). Particularly, we find experimentally that regardless of the operation frequency (128 GHz or 143 GHz), a THz signal launched into the Input port 1 of a directional coupler (see Fig. S14) is forwarded mainly into the Output port 1 (a would-be Through port in the corresponding ADM circuit) as seen in Figs. S14(a) and S14(b). At the same time, only a negligible portion of power is redirected into the Input port 2 (a would-be Drop port in the corresponding ADM circuit) as seen from Figs. S14(c) and S14(d). A small signal in the Input port 2 of a directional coupler circuit is only slightly above the THz communication system noise level (Fig. S14(e)) and is due to crosstalk between two branches of the Y-splitter. This is in stark contrast with the experimental behavior of the ADM circuits shown in Fig. 5, where only a signal at 128 GHz (grating passband) reaches the Through port, while the signal at 143 GHz (grating stopband) is completely blocked from the Through port and redirected into the Drop port.
Moreover, due to the broadband operation of the directional coupler (see Fig. 3(c)), similar BER performance characteristics (see Fig. S14(f)) are observed at the Output port 1 of the directional coupler for the two frequencies. In fact, the BER curves presented in Fig. S14(f) are very similar to those of a Through action for the complete ADM circuits presented in Fig. 6(c). This is easy to rationalize as the Through action of an ADM circuit is designed for operation outside of the grating stopband, thus being only affected by the directional coupler performance.

Supplementary Note 11: Signal-to-noise ratio (SNR) in the THz ADM circuits

Fig. S14(e) shows the eye diagram for the noise level (no signal) of the THz communication system. Due to the relatively high insertion losses for both the Add/Drop actions and Through action, the low value of SNR results in the failure of the error-free data transmission when using a photonics-based THz communication system for ADM characterization. As the BER depends exponentially on SNR (BER~exp(-SNR)), and insertion losses of the Add/Drop action are significantly higher than those of a Through action, it is expected that the BER for channel Add/Drop to be much higher than that for the channel Through. In fact, comparing eye diagrams of the THz signals at 1 Gbps propagating through the ADM (see Fig. 6(b)) with that of the noise level (see S15(e)), the SNR for channel Drop and Through actions are estimated at ~5 dB and ~11 dB respectively. From this, we expect that BER for the Add/Drop action should be several thousand times higher than that for the Through action, which is in good agreement with the experimental result shown in Fig. 6.

Supplementary Note 12: Influence of the ADM component dispersion on data transmission

To quantify the effect of the link dispersion on the maximal supported bit rate, we consider second-order modal dispersion β_2. The maximum bit rate 'B' (for ASK modulation) supported by the waveguide of a length 'L' can be estimated using Eq. (S3), which is derived by requiring that ~95% of the power of the broadened pulse form remains within the time slot allocated to logical "1" [24]:

$$B = \frac{1}{4\sqrt{|\beta_2| L}} \quad \text{(S3)}$$

In [4] it was demonstrated that in the 120-160 GHz spectral range, the two-wire waveguides (same as used in the ADMs of this paper) feature low GVD which are < 3 ps/(THz*cm) for the straight waveguide and <10 ps/(THz*cm) for the bent waveguide with 4 cm bending radius. Thus, according to Eq. (S3), dispersion of the 12.5cm-long straight In-Through waveguide will only affect data rates exceeding 40 Gbps. It is noted that in this estimate we assumed that the dispersion of a grating in the passband is similar to that of a straight waveguide, which is a valid assumption for the operational frequency far away from the edges of the grating stopband.

In contrast, for the Add/Drop action in the grating stopband, the reflective properties of the Bragg grating vary greatly within the stopband. Ultimately, it is the spectral size of the grating stopband that limits the signal bandwidth. Furthermore, due to fracturing of the stopband because of the fabrication imperfections and various interference effects (see Figs. S10(a) and S10(b)), signal bandwidth for the Add/Drop action in our ADMs is expected to be limited to ~2-4 GHz.

Supplementary Note 13: Characterization of the two-wire ADM circuits using a THz communication system

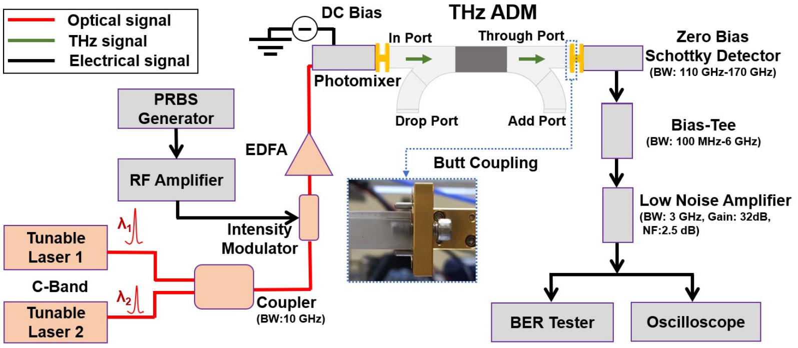

Fig. S15 Schematic of the photonics-based THz communication system to characterize THz ADM circuits. Blue dotted box: Butt coupling of the THz ADM circuit and a WR6.5 waveguide connected to the Schottky detector.

Supplementary Note 14: Numerical simulations of the two-wire waveguide Bragg gratings

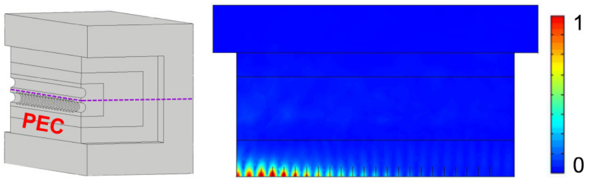

Fig. S16 Numerical simulation of two-wire WBGs. (a) The 3D model in the COMSOL Multiphysics to study WBGs. (b) The electric field distribution of THz light within grating stopband (140 GHz) in a two-wire WBG on the plane marked by the violet dashed line in Fig. S16(a)) when the THz light is launched using port boundary condition at the input facet of the WBG.

References

1. Markov, A., Guerboukha, H. & Skorobogatiy, M. Hybrid metal wire–dielectric terahertz waveguides: challenges and opportunities. *JOSA B* **31**, 2587-2600 (2014).
2. Mridha, M. K. et al. Active terahertz two-wire waveguides. *Opt. Express* **22**, 22340-22348 (2014).
3. Yan, G. et al. Low-loss terahertz waveguide Bragg grating using a two-wire waveguide and a paper grating. *Opt. Lett.* **38**, 3089-3092 (2013).
4. Cao, Y., Nallappan, K., Guerboukha, H., Xu, G. & Skorobogatiy, M. Additive manufacturing of highly reconfigurable plasmonic circuits for terahertz communications. *Optica* **7**, 1112-1125 (2020).
5. Jia, S. et al. 0.4 THz photonic-wireless link with 106 Gb/s single channel bitrate. *J. Light. Technol.* **36**, 610-616 (2018).
6. Jia, S. et al. Integrated dual-DFB laser for 408 GHz carrier generation enabling 131 Gbit/s wireless transmission over 10.7 meters. *in Optical Fiber Communication Conference* (2019), Th1C-2.
7. Liu, K. et al. 100 Gbit/s THz photonic wireless transmission in the 350-GHz band with extended reach. *IEEE Photon. Technol. Lett.* **30**, 1064-1067 (2018).
8. Karl, N. J., McKinney, R. W., Monnai, Y., Mendis, R. & Mittleman, D. M. Frequency-division multiplexing in the terahertz range using a leaky-wave antenna. *Nat. Photon.* **9**, 717-720 (2015).
9. Ma, J., Karl, N. J., Bretin, S., Ducournau, G. & Mittleman, D. M. "Frequency-division multiplexer and demultiplexer for terahertz wireless links. *Nat. Commun.* **8**, 1-8 (2017).
10. Yata, M., Fujita, M. & Nagatsuma, T. Photonic-crystal diplexers for terahertz-wave applications. *Opt. Express* **24**, 7835-7849 (2016).
11. Withayachumnankul, W., Fujita, M. & Nagatsuma, T. Integrated silicon photonic crystals toward terahertz communications. *Adv. Opt. Mater.* **6**, 1800401 (2018).
12. Reichel, K. S. et al. Electrically reconfigurable terahertz signal processing devices using liquid metal components. *Nat. Commun.* **9**, 1-6 (2018).
13. Headland, D., Withayachumnankul, W., Fujita, M. & Nagatsuma, T. Gratingless integrated tunneling multiplexer for terahertz waves. *Optica* **8**, 621-629 (2021).
14. Poulin, M., Giannacopoulos, S. & Skorobogatiy, M. Surface Wave Enhanced Sensing in the Terahertz Spectral Range: Modalities, Materials, and Perspectives. *Sensors* **19**, 5505 (2019).
15. Liu, M. et al. A graphene-based broadband optical modulator. *Nature* **474**, 64-67 (2011).
16. Kampfrath, T., Perfetti, L., Schapper, F., Frischkorn, C. & Wolf, M. Strongly coupled optical phonons in the ultrafast dynamics of the electronic energy and current relaxation in graphite. *Phys. Rev. Lett.* **95**, 187403 (2005).
17. Wang, Z. et al. Recent progress in terahertz modulation using photonic structures based on two-dimensional materials. *InfoMat* **3**, 1110-1133 (2021).
18. Chen, M., Sheng, P., Sun, W. & Cai, J. A symmetric terahertz graphene-based hybrid plasmonic waveguide. *Opt. Commun.* **376**, 41-46 (2016).
19. Xu, W. et al. Toward integrated electrically controllable directional coupling based on dielectric loaded graphene plasmonic waveguide. *Opt. Lett.* **40**, 1603-1606 (2015).
20. Riziotis, C. & Zervas, M. N. Novel full-cycle-coupler-based optical add-drop multiplexer and performance characteristics at 40-Gb/s WDM networks. *J. Light. Technol.* **21**, 1828 (2003).
21. Riziotis, C. & Zervas, M. N. Performance comparison of Bragg grating-based optical add–drop multiplexers in WDM transmission systems. *IEE P-Circ. Dev. Syst.* **149**, 179-186 (2002).
22. Roggenbuck, A. et al. Coherent broadband continuous-wave terahertz spectroscopy on solid-state samples. *New J. Phys.* **12**, 043017 (2010).
23. Cao, Y., Nallappan, K., Guerboukha, H., Gervais, T. & Skorobogatiy, M. Additive manufacturing of resonant fluidic sensors based on photonic bandgap waveguides for terahertz applications. *Opt. Express* **27**, 27663-27681 (2019).
24. Agrawal, G. P. Lightwave technology: telecommunication systems. John Wiley & Sons, 2005.
